# Supplementary material for: WordCommentsAnalyzer: A windows software tool for qualitative research
Source: F1000Res. 2018 Sep 4;7:536. Originally published 2018 May 3. [Version 2] doi: 10.12688/f1000research.14819.2 (PMC6137411; doi:10.12688/f1000research.14819.2)
Supplement: Supplementary file 3 [file f1000research-7-17614-s0002.tgz › b2a79496-4ada-47c4-9682-a99ce867836d.zip!/Eight Iranian Health Journals/Yafteh _43.docx]

© Yafteh  . This work is licensed under CC BY 4.0 (https://creativecommons.org/licenses/by/4.0/).

Volume & Issue: دوره 10 - شماره 2

Title: بررسی رابطه عفونت کلامیدیا پنومونیه و هلیکوباکترپیلوری با آترواسکلروزیس

http://yafte.lums.ac.ir/article-1-74-fa.html

مقدمه: آترواسکلروزیس شایعترین علت مرگ در کشورهای پیشرفته بوده و عامل حدود یک میلیون مرگ در سال درایالات متحده می باشد. علاوه بر عوامل شناخته شده، عوامل عفونی را در ایجاد آن دخیل دانسته اند و هدف از مطالعه ما، کشف ارتباط این عوامل (کلامیدیا پنومونیه و هلیکوباکتر پیلوری) با آترواسکلروزیس می باشد. مواد و روش‌ها: در این مطالعه که ازنوع تحلیلی و Case-Control (مورد- شاهدی) می باشد تعداد 30 مورد بیمارکه تحت عمل جراحی بای پس عروق کرونر قرار گرفته هم از نظر سرولوژی وهم ازنظر هیستولوژی از نمونه های بافتی محل پانچ آئورت و30 مورد شاهد که تحت آنژیوگرافی قرارگرفته وعروق کرونر سالم دارند فقط از نظر سرولوژی مورد بررسی قرار گرفته و یافته ها به روشهای آماری تجزیه و تحلیل شدند. یافته‌ها: سرولوژی دو گروه از لحاظ IgA و IgG ضد Cpn و IgG ضد Hp با هم تفاوت آماری معنی داری نداشتند ولی IgA ضد Hp در گروه بیمار بطور معنی داری از گروه کنترل دارای موارد مثبت بیشتری بود (003/0 p< ). در ضمن در گروه بیمار در پانچ بیوپسی از جدار ائورت 6 مورد باکتری Cpn (20%) و 8 مورد باکتری Hp (7/26%) یافت شد و وقتی سرولوژی گروه بیمار با پاتولوژی جدار آئورت با هم مقایسه شد دیده شد که رفتار دوباکتری درتهاجم موضعی و بروز واکنش سیستمیک با هم متفاوت می باشد . بحث و نتیجه‌گیری: این مطالعه نشان داد که Hp ممکن است در بروز واکنش سیستمیک جهت ایجاد آترواسکلروزیس موثر باشد ولی نقش Cpn را رد می کند ولی هردو باکتری قادر به تهاجم موضعی به جدار آئورت هستند.

Title: بررسی شیوع باکتری های بی هوازی گرم منفی در بیماران مبتلا به پریودنتیت مزمن

http://yafte.lums.ac.ir/article-1-75-fa.html

مقدمه: پریودنتیت یک بیماری عفونی شایع والتهابی است که باعث تخریب بافت­ های نگهدارنده دندان و از دست رفتن دندان می­شود . یافته­های اخیر نشان می­دهد که عفونت پریودنتال به طرز قابل توجهی باعث افزایش خطر ابتلا به بعضی از بیماری­های سیستمیک می­گردد. از مهمترین عوامل ایجاد عفونت در پریودنشیوم، باکتری­ها به ویژه پورفیروموناس ژنژیوالیس و باکتروئیدس فورسیتوس می­باشند.در بین روش­های تشخیص باکتری­ها به نظر می ­رسد Multiplex PCR از حساسیت و ویژگی بالایی برخوردار است با این روش می­توان چند نوع میکروارگانیسم را به طور همزمان با هم تشخیص داد. مواد و روش‌ها: نمونه پلاک زیر لثه­ای از 61 بیمار مبتلا به پریودنتیت مزمن (34 زن، 27 مرد، دامنه سنی 69-24 سال، میانگین سنی 43 سال) و 40 فرد سالم (22 زن، 18 مرد، دامنه سنی 69-21 سال، میانگین سنی 41 سال)که به بخش پریودنتیکس دانشکده دندانپزشکی دانشگاه علوم پزشکی اصفهان مراجعه کرده بودند، گرفته شد.سپس DNA نمونه­ها استخراج و Multiplex PCR انجام شد. یافته‌ها: باکتروئیدس فورسیتوس 50/52 % از بیماران مبتلا به پریودنتیت مزمن تشخیص داده شد در حالی که در هیچ سایت سالمی دیده نشد و پورفیروموناس ژنژیوالیس در 61/83 % از بیماران مبتلا به پریودنتیت مزمن و 40% از افراد سالم تشخیص داده شد. (05/0 p < ) . بحث و نتیجه‌گیری: به کمک Multiplex PCR می­توان این دو باکتری را به صورت همزمان تشخیص دادو نتایج بدست آمده نشان می­دهد پورفیروموناس ژنژیوالیس از اهمیت بیشتری در ایجاد پریودنتیت مزمن برخوردار است . با توجه به این که بیماری پریودنتیت یکی از عوامل مستعد کننده بیماری­های سیستمیک است تشخیص و درمان به موقع این بیماری حائز اهمیت می­باشد .

Title: بررسی ارتباط بین وضع تغذیه کودکان زیر دو سال مبتلا به گاستروانتریت با طول مدت بستری آنها در بیمارستان

http://yafte.lums.ac.ir/article-1-76-fa.html

مقدمه: مطالعات متعدد حاکی از آن است که اسهال در مبتلایان به سوء تغذیه شایع تر است، زیرا سوء تغذیه زمینه ساز عفونت شده و عفونت نیز منجر به اسهال می شود که خود چرخه معیوب شناخته شده ای است با توجه به شیوع بالای گاستروانتریت در کودکان، و تاثیر وضعیت تغذیه ای بر بروز عفونتهای تنفسی و گوارشی، همچنین هزینه های بالای وارده بر دولت و خانواده ها به دلیل دوره های بستری طولانی مدت کودکان، این تحقیق به منظور بررسی ارتباط بین وضعیت تغذیه ای کودکان مبتلا به گاستروانتریت با طول مدت بستری آنها، در بیمارستان شهید مدنی خرم آباد در سال 1386 انجام گرفت. مواد و روش‌ها: این تحقیق به صورت مقطعی بر روی 100 کودک زیر 2 سال مبتلا به گاستروانتریت انجام گرفت بیماران به دو گروه 50 نفری، شامل کودکان با «وضع تغذیه مناسب» و کودکان مبتلا به «سوء تغذیه» تقسیم شدند. جهت از بین بردن متغیرهای مخدوشگر، کودکان مورد مطالعه با داشتن : وزن هنگام تولد بالاتر از 2500 گرم، فاصله تولد با نوزاد قبلی بیشتر از 2 سال، نداشتن سابقه بستری به علت هرگونه بیماری در 6 ماهه گذشته و محدوده سنی مادران بین 20 تا 35 سال، به روش متوالی انتخاب شده و اطلاعات مورد نیاز از طریق پرسشنامه جمع آوری گردید میانگین مدت بستری در دو گروه فوق مقایسه گردید و تاثیر متغیرهای مداخله گر احتمالی با فاصله اطمینان 95 درصد تعیین گردید. یافته‌ها: در این مطالعه بین میانگین مدت بستری (بر حسب روز) با وضع تغذیه، در دو گروه کودکان مورد مطالعه تفاوت معنی دار آماری مشاهده گردید (001/0 p< )، بطوریکه متوسط مدت بستری در کودکان زیر 2 سال مبتلا به گاستروانتریت توام با «سوء تغذیه»، 86/3 روز بود، حال آنکه در کودکان با «وضع تغذیه مناسب» 58/2 روز بود از بین متغیرهای مخدوشگر موثر بر طول مدت بستری، فقط، تغذیه انحصاری با شیر مادر بر طول مدت بستری تاثیر آماری معنی داری داشت (001/0 < p ) . بحث و نتیجه‌گیری: با توجه به نتایج به دست آمده مشخص شد که درمیان کودکان مبتلا به گاستروانتریت طول مدت بستری در بیمارستان ارتباط معنی داری با وضع تغذیه آنان دارد.

Title: بررسی وضعیت فشار خون در زنان روستایی استان کرمان و ارتباط آن با نمایه های تن سنجی

http://yafte.lums.ac.ir/article-1-77-fa.html

مقدمه: فشار خون بالا یکی از عوامل خطرساز در بروز بیماریهای قلبی- عروقی است. از طرفی، نمایه های تن سنجی مانند نمایه توده بدن ( BMI )، محیط دور کمر و نسبت دور کمر به دور باسن ( WHR ) بطور مستقل باعث تغییرات فشار خون می شوند. هدف از مطالعه حاضر بررسی وضعیت فشار خون در زنان روستایی استان کرمان و تعیین ارتباط آن با نمایه های تن سنجی بود. مواد و روش‌ها: در این مطالعه مقطعی 370 نفر از زنان سنین باروری (45-20 سال) ساکن روستاهای استان کرمان به روش نمونه گیری تصادفی خوشه ای انتخاب شدند. اطلاعات عمومی افراد مورد بررسی با استفاده از پرسشنامه و مصاحبه مستقیم جمع آوری شد. اندازه های تن سنجی شامل قد، وزن، محیط دور کمر و دور باسن اندازه گیری و نمایه توده بدن ( BMI ) و نسبت دورکمر به دور باسن ( WHR ) برای هر یک از افراد مورد بررسی محاسبه گردید. تجزیه و تحلیل آماری داده ها با استفاده از آزمونهای آماری آنالیز واریانس یکطرفه (ANOVA) ، آزمون t مستقل و ضریب همبستگی پیرسون انجام شد. یافته‌ه ا: 9/15 درصد و 8/22 درصد زنان مورد بررسی به ترتیب مبتلا به چاقی و چاقی شکمی (8/0< WHR ) بودند. همچنین، 3/14 درصد از زنان، مبتلا به پرفشاری خون بودند. فشار خون زنان با افزایش وزن، BMI ، WHR ، محیط دور کمر و تعداد بارداریها بطور معنی داری افزایش یافته بود. علاوه بر این، بین BMI با فشارخون سیستولی ( SBP ) و فشار خون دیاستولی ( DBP ) همبستگی مثبت معنی داری وجود داشت ( به ترتیب 0001/0> p ، 32/0= r و002/0> p ، 26/0= r ). همچنین، WHR با SBP (003/0> p ، 22/0= r ) و DBP (002/0> p ، 24/0= r ) همبستگی مثبت معنی دار داشت. محیط دور کمر نیز با DBP همبستگی مثبت معنی دار داشت (05/0> p ، 18/0= r ). بحث و نتیجه‌گیری: بین نمایه های تن سنجی با تغییرات فشار خون در زنان سنین باروری ارتباط مستقیم معنی دار وجود دارد. بطور کلی کنترل وزن و حفظ تناسب اندام در پیشگیری از ابتلای زنان به پرفشاری خون مفید خواهد بود.

Title: حاملگیهای پرخطر ناشی از تروماهای جسمی درزنان باردار مراجعه کننده به مرکز پزشکی قانونی و مراکز درمانی دانشگاهی شهر خرم آباد

http://yafte.lums.ac.ir/article-1-78-fa.html

مقدمه: واردشدن ترومابه زنان باردارمی تواند منجر به عوارض زودرس مانند دکولمان جفت و زایمان زودرس و در نهایت خونریزی و مرگ مادر و جنین شود. به همین دلیل تروما یکی از علل مهم بروز حاملگیهای پرخطرمحسوب می شود. از هر 12 زن باردار یک نفر دچار صدمات جسمی می شود و 7% حاملگی ها گرفتار عوازض ناشی از صدمات می شوند. سوانح رانندگی معمولترین علت غیر زایمانی مرگ و میر مادر در دوران بارداری است. درهمین راستا پژوهشی با هدف تعیین حاملگیهای پر خطر ناشی از تروماهای جسمی در زنان باردار مراجعه کننده به مرکز پزشکی قانونی ومراکز درمانی دانشگاهی شهر خرم آباد انجام شد. مواد و روش‌ها: پژوهش حاضر یک مطا لعه توصیفی و مقطعی است. در این پژوهش کلیه زنان بارداری (71 نفر ) که به علت صدمات ازاول مهر1384 تا پایان شهریور1385( به مدت یکسال) به مرکز پزشکی قانونی ومراکز درمانی دانشگاهی خرم آبادمراجعه کرده بودند مورد بررسی قرارگرفتند. ابزار گرداوری داده های پژوهش یک پرسشنا مه و برگ ثبت مشاهدات بود وکلیه واحدهای مورد پژوهش ظرف 24 تا 48 ساعت اول بعد از وقوع تروما در محل بستری مورد بررسی قرار میگرفتند و پس ازترخیص یک ماه بعداز سانحه بر اساس آدرس محل سکونت از نظر وقوع عوارض پیگیری انجا م می شد.اطلا عات به دست امده با استفاده از نرم افزار SPSS مورد تجزیه وتحلیل قرارگرفت وجهت تجزیه و تحلیل داده ها از آمار تو صیفی واستنباطی استفاده شد. یافته‌ها: براساس نتایج پژوهش علت مراجعه 1/60% ازواحد های مورد پژوهش تروماهای اتفاقی و 6/31 %سورفتار و 3/8% خود کشی بود که شایعترین علت تروماهای اتفاقی تصادف با وسایل نقلیه بود.از نظر تروماهای عمدی زنانی که مورد سورفتار همسر قرار گرفته بودند دارای بیشترین فراوانی بودندمیانگین سن بارداری 6/22 هفته ونیزبیشترین محل آسیب ناحیه شکم و اکثر صدمات به تروما های متعدد اختصاص داشت.بیشترین عارضه در تروماهای اتفاقی زایمان زودرس و درصدمات عمدی مرگ مادر و جنین بود.در این پژوهش آزمونهای آماری ارتباط معنی داری بین علت صدمات ونوع تروما با عوارض بارداری نشان نداد. بحث و نتیجه‌گیری: نتایج پژوهش حاکی از شیوع بالای سوانح و سورفتار وخودکشی در زنان باردارموردپژوهش است وکلیه عوارض ایجادشده عوارض زودرس می باشد همچنین مقایسه نتایج با سایر پزوهشهای مشابه حاکی از وقوع بیشتر صدمات عمدی است که با آموزش به مادران و خانواده های آنان و آگاه ساختن از عوارض جسمی وروانی سو رفتار و نیزجلوگیری از عدم قرارگیری مادران در شرایط پر خطرمانند بستن کمربند ایمنی میتوان تا حد موثری از وقوع سوانح و عوارض بالاخص مرگ مادر و جنین پیشگیری نمود.

Title: مقایسه اثر لیزر کم ‌توان با اولتراسوند درمانی در درمان تاندونیت کلاهک چرخاننده

http://yafte.lums.ac.ir/article-1-80-fa.html

مقدمه: درد شانه سومین اختلال اسکلتی‌عضلانی بعد از دردهای کمر و گردن است. بیشتر علائم شانه به تاندون کلاهک چرخاننده نسبت داده شده است. این مطالعه به منظور مقایسه اثرات لیزر کم‌توان با اولتراسوند درمانی در درمان تاندونیت کلاهک چرخاننده شانه انجام شد . مواد و روش‌ها: این کارآزمایی بالینی در سال 1385، در دانشگاه علوم پزشکی زاهدان انجام شد. سی بیمار با تشخیص تاندونیت کلاهک چرخاننده به صورت تصادفی در دو گروه لیزر کم‌توان (15 بیمار) و اولتراسوند درمانی (15 بیمار) قرار گرفتند. قدرت عضلات ابداکتور، چرخاننده‌های داخلی و خارجی شانه با دینامومتر دستی (کیلوگرم) و دامنه حرکات ابداکشن، چرخش داخلی و خارجی شانه با گونیامتر (درجه) قبل و بعد از درمان اندازه‌گیری شد. درد با مقیاس اندازه‌گیری دیداری (VAS) ارزیابی شد. گروه لیزر کم‌توان به مدت 3 دقیقه تحت درمان با لیزر کم توان گالیوم-آرسناید، فرکانس 5 کیلوهرتز، و پروب با توان متوسط خروجی 100میلی وات، طول موج 905 نانومتر، پهنای پالس 200 نانوثانیه و میزان انرژی 6 ژول و گروه اولتراسونددرمانی به مدت 10 دقیقه در روز تحت درمان با اولتراسوند نوع منقطع (1:4) با شدت 1 وات بر سانتی‌متر مربع و فرکانس 1 مگاهرتز قرار گرفتند. درمان برای هر دو گروه 3 روز در هفته و به مدت 10 روز انجام شد. داده‌ها با آزمون‌های آماری t زوج و t مستقل تجزیه و تحلیل شدند . یافته‌ها: در گروه لیزر کم‌توان درجه درد در حرکت ابداکشن شانه از 6/1 ± 06/6 به 3/1 ± 5، در حرکت چرخش داخلی شانه از 5/1 ± 3/5 به 3/1 ± 7/4 و در حرکت چرخش خارجی شانه از 4/1 ± 06/5 به 44/1 ± 3/4 کاهش یافت (05/0> p ). در گروه اولتراسوند درمانی درجه درد در حرکت ابداکشن شانه از 06/1 ± 3/6 به 01/1 ± 2/6، در حرکت چرخش داخلی شانه از 9/0 ± 07/6 به 84/0 ± 9/5 و در حرکت چرخش خارجی شانه از 92/0 ± 87/5 به 96/0 ± 8/5 کاهش یافت (05/0< p ). مقایسه نتایج بعد از درمان بین دو گروه نشان داد که قدرت عضلات ابداکتور، چرخاننده‌های داخلی و خارجی شانه در گروه لیزر کم‌توان نسبت به گروه اولتراسوند درمانی ارتقا (05/0> p ) و درجه درد در هر سه حرکت در گروه لیزر نسبت به گروه اولتراسوند درمانی کاهش یافته است (05/0> p ) . بحث و نتیجه‌گیری: نتایج مطالعه از کاربرد یک دوره لیزر کم‌توان گالیوم-آرسناید به منظور کاهش درد، و در نتیجه بروز قدرت عضلات، در بیماران مبتلا به تاندونیت کلاهک چرخاننده حمایت می‌کنند. یک دوره درمان با لیزرکم‌توان موثرتر از اولتراسوند درمانی است .

Title: بررسی نظرات وآگاهی پرستاران دررابطه بااشتباهات دارویی وراههای پیشگیری ازآن دربیمارستان های آموزشی خرم آباد1384

http://yafte.lums.ac.ir/article-1-81-fa.html

مقدمه: اشتباهات داروئی شایع ترین علت قابل اجتناب آسیب با منشا ناشناخته برای بیماران هستند و از هر3 حادثه داروئی یک مورد در اثر اشتباه پرستار هنگام دادن دارو به بیمار اتفاق می افتد. از آنجا که دستورات داروئی بخش مهمی از فرایند درمان بیمار می باشد، عدم توجه به اصول صحیح به کارگیری آن می تواند منجر به بروز مشکلات عدیده ای چون درمان نادرست و ناموفق یا ناقص و بالا خره بروز معضلات قانونی گردد. هدف از مطالعه حاضر تعیین نظرات و آگاهی پرسنل پرستاری در رابطه با اشتباهات دارویی و راههای پیشگیری از آن در بیمارستان های آموزشی خرم آباد در سال 1384 بود. مواد و روش‌ها: این تحقیق یک مطالعه توصیفی-مقطعی است. نمونه پژوهش شامل 86 پرستار شاغل در بیمارستان های آموزشی خرم آباد در سال 1384 بود که به روش تصادفی انتخاب شدند. ابزار جمع آوری اطلاعات پرسشنامه و روش جمع آوری اطلاعات مصاحبه ساختار یافته بود. اطلاعات جمع آوری شده با استفاده از نرم افزار SPSS نسخه13 و آزمونهای آمار توصیفی و مجذور کای مورد بررسی و تجزیه تحلیل قرار گرفت. یافته‌ها: نتایج نشان داد پرستاران کمبود نیرو (100%)، زیاد بودن شیفت، شب کاری و شیفت های طولانی و پشت سر هم (7/83%)، مشکلات فردی یا شخصی پرستار (9/79%)، حضور همراه و شلوغی بخش (9/79%) و شرایط نامناسب محیطی (3/73%) را مهمترین علل وقوع اشتباهات دارویی گزارش کردند. ترس از توبیخ و تنبیه (4/88%)، مهم نبودن اشتباه دارویی (57%)و عدم برخورد حمایتی مسولان پرستاری (50%) مهمترین علل عدم گزارش اشتباهات دارویی گزارش شدند. افزایش نیرو متناسب با تعداد بیماران (8/98%)، آموزش پرسنل (5/96%) و اطلاع رسانی در مورد داروهای جدید (8/69%) مهمترین روشهای پیشگیری از بروز اشتباهات دارویی گزارش شدند. در 8/62% میزان آگاهی پرستاران در مورد اشتباهات دارویی و روشهای پیشگیری از آن در حد متوسط بود. بحث و نتیجه‌گیری: نتایج نشان داد از نظر پرستاران اقداماتی مثل کاهش فشار کار و افزایش نیرو متناسب با تعداد بیمار، بهبود شرایط محیطی بخش ها و حذف مخدوش کننده های تمرکز، انجام اقداماتی برای آموزش و بهبود آگاهی پرسنل، افزایش نقش فارماکولوژیستهای بالینی، ایجاد سیستم گزارش خطاهای رخ داده و تمرکز بر علت بروز خطانه فردیکه مرتکب اشتباه شده و افزایش سیستم های کنترل و نظارت می تواند در کاهش خطاهای دارویی موثر باشد و باید برای اقدامات و قوانین مربوط به پیشگیری و گزارش اشتباهات دارویی هم به فرد و هم بهسیستم توجه داشت.

Title: بررسی تأ ثیر وضعیتهای دمر، به پشت خوابیده و در آغوش مادر (تماس پوستی مادر با نوزاد) بردرد نوزادان حین خونگیری وریدی

http://yafte.lums.ac.ir/article-1-82-fa.html

مقدمه: نوزادان حتی وقتی سالم متولد می شوند ممکن اسن تحت عملیات دردناک قرار گیرند. کاهش درد حین مداخلات پزشکی حق هر موجود زنده ای می باشد. در بسیاری از مطالعات ثابت شده است که تجربیات دردناک دوران نوزادی علاوه بر عوارض زودرس از قبیل تاکیکاردی، تاکی پنه و افزایش نیازهای متابولیک بدن می تواند پاسخ های رفتاری و فیزیولوژیک فرد نسبت به درد را در دوره های بعدی زندگی تشدید نماید. هدف از انجام این مطالعه بررسی تاثیر وضعیتهای مختلف نوزادان بر روی پاسخ های آنان به درد درحین خونگیری وریدی می باشد. مواد و روش‌ها: این مطالعه از نوع کارآزمایی بالینی است.جامعه مورد مطالعه شامل نوزادان بستری به دلیل زردی فیزیولوژیک در بیمارستان کودکان خرم آباد در نیمه دوم سال 1384 بودند که هیچگونه بیماری زمینه ای دیگری نداشتند. نوزادان به جززردی از لحاظ کلیه معاینات سیستمیک طبیعی بودند. حجم نمونه 50 نفر بوده، ازهمه نوزادان در طی سه روز متوالی در سه وضعیت مختلف دمر (Supine) ، به پشت خوابیده (Prone) و در آغوش مادر (Kangaroo Care) و از مکانهای مشابه بدن نوزاد خونگیری وریدی به عمل آمد. جهت بررسی پاسخ های نوزادان به درد از معیار DAN استفاده شد. پس از ثبت اطلاعات مورد نیاز اعم از جنسیت، وضعیت ترم، سن و وزن، داده ها با استفاده از نرم افزار آماری SPSS و تستهای آماری کولموگروف – اسمیرنوف، فریدمن، ویلکاکسون، همبستگی و T مورد تجزیه و تحلیل قرار گرفتند. یافته‌ها: براساس نتایج بدست آمده مشخص گردید که کمترین پاسخ نوزادان به درد در وضعیت KC یعنی حالتی که نوزاد در آغوش مادر بوده و تماس پوستی مستقیم وجود دارد می باشد. پاسخ های ضد درد دروضعیت Supine بیشترین مقدار رانشان داد (001/0 p< ).همچنین مشخص گردید که بین میزان انواع پاسخ های ضد درد در نوزادان (تغییرات صورت، حرکات اندامها، پاسخ های صوتی) در سه پوزیشن مورد مطالعه، در تمامی موارد مقایسه از نظر آماری اختلاف معنی داری وجود دارد. بحث و نتیجه‌گیری: با توجه به یافته های این مطالعه، وضعیت KC در کاهش درد وی حین فرآیند های دردناک می تواند مؤثرتر از دیگر وضعیتها باشد. جهت تکمیل نتایج این مطالعه توصیه می شود که مطالعات مشابهی صورت بگیرد.

Title: گزارش یک مورد نادر توده تیروئید نابجا در مدیاستن قدامی

http://yafte.lums.ac.ir/article-1-83-fa.html

مقدمه: گواترهای نابجای اولیه مدیاستن بسیار نادر بوده و کمتر از 1% گواترهای خلف جناغی و مدیاستینال است و خونگیری آنها از عروق داخل قفسه صدری می باشد. گزارش مورد: ما یک بیمار مرد 61 ساله با گواتر مولتی ندولر در گردن و گواتر نابجای اولیه مدیاستن را معرفی می کنیم که با یک توده بزرگ در مدیاستن قدامی که از حدود 6 ماه قبل از مراجعه با علامت تنگی نفس و خس خس سینه مراجعه نموده بود که با برش عرضی گردن و استرنوتومی نسبی جدار باز شد و توده های مزبور برداشته شدند و تمامی علائم بالینی بیمار در پیگیری از بین رفت. خونگیری توده مدیاستن از عروق داخل قفسه صدری شریان و ورید بی نام بود . بحث و نتیجه‌گیری: ماتیرویدنابجای مدیاستن واقعی را گزارش نمو دیم که با برش مید استرنوتو می به صورت کامل برداشته شد و کلیه علایم بیمار از بین رفت .
